# Different Regulatory Modes of *Synechocystis* sp. PCC 6803 in Response to Photosynthesis Inhibitory Conditions

**DOI:** 10.1128/mSystems.00943-21

**Published:** 2021-12-07

**Authors:** Sang-Hyeok Cho, Yujin Jeong, Seong-Joo Hong, Hookeun Lee, Hyung-Kyoon Choi, Dong-Myung Kim, Choul-Gyun Lee, Suhyung Cho, Byung-Kwan Cho

**Affiliations:** a Department of Biological Sciences, Korea Advanced Institute of Science and Technology, Daejeon, Republic of Korea; b Department of Biological Engineering, Inha University, Incheon, Republic of Korea; c Institute of Pharmaceutical Research, College of Pharmacy, Gachon University, Incheon, Republic of Korea; d College of Pharmacy, Chung-Ang University, Seoul, Republic of Korea; e Department of Chemical Engineering and Applied Chemistry, Chungnam National University, Daejeon, Republic of Korea; f Innovative Biomaterials Center, KI for the BioCentury, Korea Advanced Institute of Science and Technology, Daejeon, Republic of Korea; Scripps Institution of Oceanography

**Keywords:** cyanobacteria, *Synechocystis*, photosynthesis, transcriptome, translatome

## Abstract

Cyanobacteria are promising industrial platforms owing to their ability to produce diverse natural secondary metabolites and nonnative value-added biochemicals from CO_2_ and light. To fully utilize their industrial potency, it is critical to understand their photosynthetic efficiency under various environmental conditions. In this study, we elucidated the inhibitory mechanisms of photosynthesis under high-light and low-temperature stress conditions in the model cyanobacterium *Synechocystis* sp. PCC 6803. Under each stress condition, the transcript abundance and translation efficiency were measured using transcriptome sequencing (RNA-seq) and ribosome profiling, and the genome-wide transcription unit architecture was constructed by data integration of transcription start sites and transcript 3′-end positions obtained from differential RNA-seq and sequencing of 3′-ends (Term-seq), respectively. Our results suggested that the mode of photosynthesis inhibition differed between the two stress conditions; high light stress induced photodamage responses, while low temperature stress impaired the translation efficiency of photosynthesis-associated genes. In particular, poor translation of photosystem I resulted from ribosome stalling at the untranslated regions, affecting the overall photosynthetic yield under low temperature stress. Our comprehensive multiomics analysis with transcription unit architecture provides foundational information on photosynthesis for future industrial strain development.

**IMPORTANCE** Cyanobacteria are a compelling biochemical production platform for their ability to propagate using light and atmospheric CO_2_ via photosynthesis. However, the engineering of strains is hampered by limited understanding of photosynthesis under diverse environmental conditions such as high-light and low-temperature stresses. Herein, we decipher the transcriptomic and translatomic responses of the photosynthetic efficiency to stress conditions using the integrative analysis of multiomic data generated by RNA-seq and ribosome profiling, respectively. Through the generated massive data, along with the guide of the genome-wide transcription unit architecture constructed by transcription start sites and transcript 3′-end positions, we identified the factors affecting photosynthesis at transcription, posttranscription, and translation levels. Importantly, the high-light stress induces photodamage responses, and the low-temperature stress cripples the translation efficiency of photosynthesis-associated genes. The resulting insights provide pivotal information for future cyanobacterial cell factories powered by the engineering toward robust photosynthesis ability.

## INTRODUCTION

Among the photoautotrophic organisms, cyanobacteria, along with algal species, are important industrial producers of third-generation biofuels and require neither farmland nor freshwater for their production (1). Their genomes include various biosynthetic gene clusters for producing various secondary metabolites, including terpenes, bioactive peptides, and other macromolecule derivatives (2). In addition, cyanobacteria have been proposed as effective production hosts for nonnative chemicals, including 2,3-butandiol, isoprene, and squalene (3–5). To determine their potential as industrial cell factories, engineering of their photosynthetic capability based on the understanding of their underlying mechanisms is crucial (6, 7).

The photosynthetic activity of cyanobacteria is susceptible to environmental factors such as illumination and temperature (8). For example, high light (HL) stress directly inhibits photosystem II (PSII) and indirectly damages photosystem I (PSI). PSII receives solar radiation and transfers electrons to the pigments to excited states using the absorbed light energy. This system is predominantly processed by the D1/D2 core protein heterodimer, and the turnover rate of the core protein increases under such conditions for repair (9). In addition, cyanobacteria reduce the antenna protein and phycobilisome, to decrease excessive amounts of electron transfer (10). The electrons are carried through the plastoquinone/plastoquinol (PQ/PQH_2_) pool may cause photodamage on PSI if the constant energy influx is not resolved, where the increased cyclic electron flow can raise the efflux rate from PSI (11, 12). Also, the detrimental effect of low-temperature (LT) stress on photosynthetic microorganisms causing growth retardation has been presented in previous studies (13). In particular, LT stress affects membrane fluidity, and resulting physiological responses are also well studied (14, 15). There have been speculations that the decrease in membrane fluidity may result in decreased electron transport, eventually crippling the photosynthesis (13). Such detrimental effects of low temperature on photosynthesis have also been studied using plant leaves and chloroplasts and reported decreased enzyme activities of the Calvin cycle and accumulation of oxidative stress in the reaction centers of the photosystems (16–18). Moreover, the repair of D1 protein, which comprises the reaction center of PSII, is decreased at LTs (19). Recent studies have suggested that photosynthesis is also inhibited by the imbalance of the membrane PQ/PQH_2_ pool in algal species under LT stress conditions (13). However, the decrease in temperature has a global effect on thermodynamics, making it difficult to pinpoint the cause of photosynthetic deterioration.

Among the diverse cyanobacterial species, *Synechocystis* sp. PCC 6803 is a model organism that has a relatively high growth rate and unicellular morphology. The availability of its genome sequence has facilitated both genome structural and protein functional studies (20). Although several transcriptomic studies have been performed to understand its responses to stress conditions, integrative analysis at transcriptional and translational levels has not been applied to high-light or low-temperature stress conditions (21–30). In this study, we measured the changes in the transcriptome and translatome of *Synechocystis* sp. PCC 6803 in response to HL or LT stress conditions. Furthermore, we comprehensively analyzed the genome-scale data based on the genomic architecture, which includes the transcription unit (TU) architecture determined from the 5′- and 3′-end information obtained from differential transcriptome sequencing (dRNA-seq) and sequencing of 3′-ends (Term-seq) (31, 32). By analyzing genomic architecture data alongside transcriptome and translatome data, we were able to investigate the posttranscriptional regulation in untranslated regions (UTRs) responsible for declining photosynthetic yield under stress conditions.

## RESULTS

### Transcriptome changes under HL and LT stress conditions.

**FIG 1:**
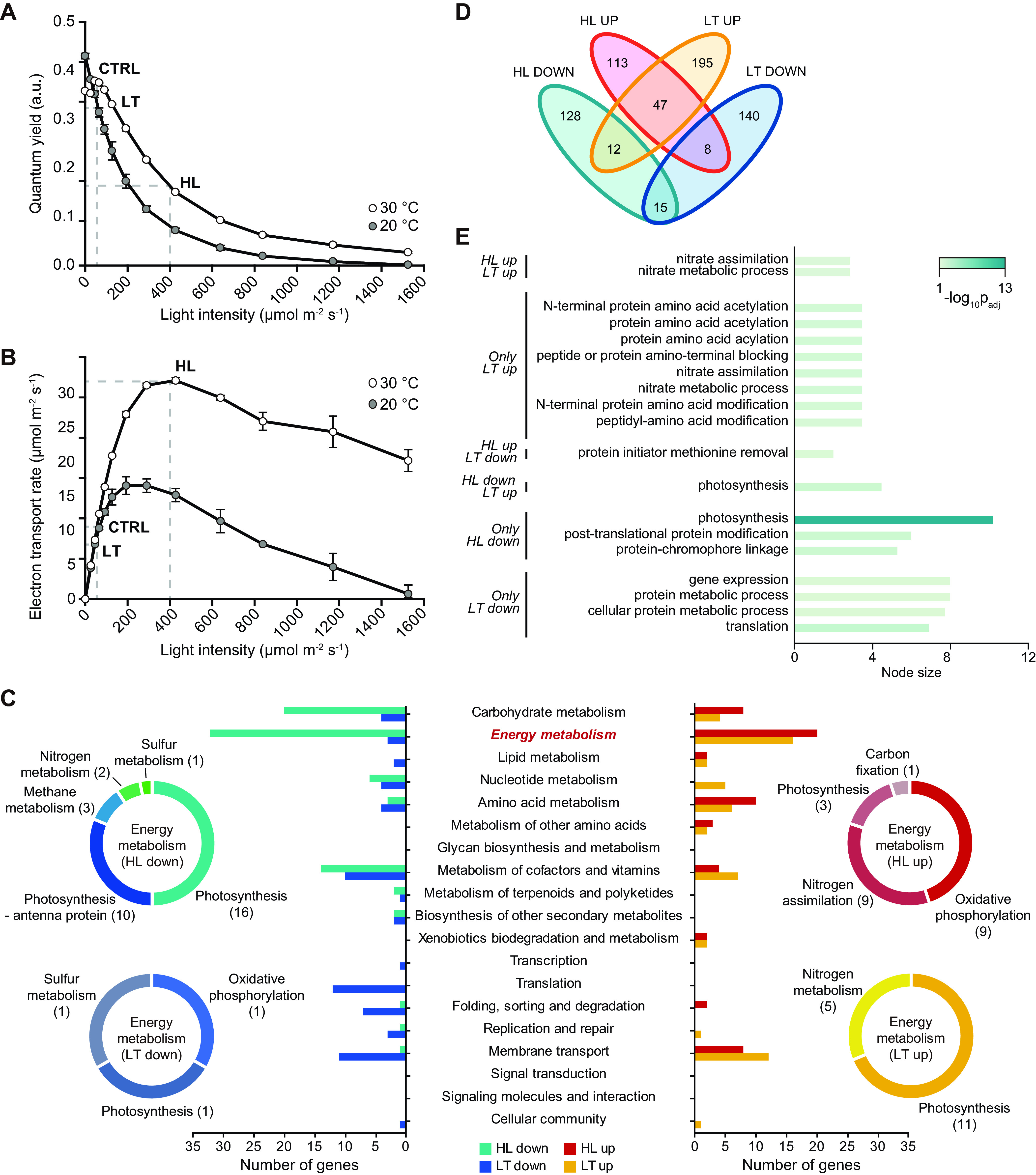
Photosynthetic efficiency and RNA-seq analysis. Quantum yield (A) and electron transport rate (B) measurements under control (30°C) and low (20°C) temperatures with various degrees of illumination. The dashed lines indicate the CTRL, HL, and LT conditions. (C) DEGs were categorized by Kyoto Encyclopedia of Genes and Genomes (KEGG) ID, and details of energy metabolism category were plotted in a pie chart. (D) DEGs were plotted as a Venn diagram (|log_2_ FC| > 1 and *P* < 0.05). (E) DEGs of each section were examined by GO term enrichment analysis. Abbreviations: a.u., arbitrary unit; CTRL, control; DEG, differentially expressed gene; GO, gene ontology; HL, high light; LT, low temperature.

We analyzed the transcriptome changes of *Synechocystis* sp. PCC 6803 under HL and LT stress conditions. To this end, we first measured cellular quantum yields and electron transport rates according to different light intensities under LT conditions (20°C) and compared these to those obtained from the normal growth temperature (30°C). The photosynthetic activity can be defined as the product of quantum yield and electron transport rate. Under LT conditions, the cell growth rate decreased, but the cells remained viable (see Fig. S1 in the supplemental material). The quantum yield decreased as light intensity increased and decreased further at 20°C than at 30°C across all light intensities (Fig. 1A). The electron transport rate of both temperatures increased until reaching plateau and decreased after plateau. Notably, the quantum yield decreased over 2-fold under an HL intensity of 400 μmol photons m^−2^ s^−1^ compared to that under control conditions (CTRL) of 50 μmol photons m^−2^ s^−1^. Based on the photosynthetic properties, we determined the following three conditions for RNA-seq: CTRL (30°C, 50 μmol photons m^−2^ s^−1^), HL (30°C, 400 μmol photons m^−2^ s^−1^), and LT (20°C, 50 μmol photons m^−2^ s^−1^) (Fig. 1A). RNA-seq generated 15.1 million to 39.5 million sequence reads mapped to the reference genome (GenBank accession no. NC_000911.1) with at least 169× coverage (Table S1). The transcriptome patterns under CTRL, HL, and LT conditions were compared using hierarchical clustering and principal-component analysis, resulting in distinctive expression patterns under different conditions (Fig. S2). RNA-seq data were then normalized by DEseq2 to compare transcript levels of individual genes, resulting in more than 3,219 genes showing a normalized expression value of 10 or higher across all growth conditions (see Table S2 at https://figshare.com/s/e10b83495a3a1fff2b1d). Next, differentially expressed genes (DEGs) under HL and LT conditions compared to the CTRL condition were determined from the normalized expression values (|log_2_ fold change [FC]| > 1; DESeq2 *P* value < 0.05). Totals of 284 and 360 DEGs were determined from the HL and LT conditions, respectively.

10.1128/mSystems.00943-21.1 FIG S1 Growth profiling under each condition. The fresh cell weight of *Synechocystis* sp. PCC 6803 was measured under the CTRL, HL, and LT conditions. The *y* axis is on a log scale. Abbreviations: CTRL, control; HL, high light; LT, low temperature. Download FIG S1, EPS file, 1.3 MB. Copyright © 2021 Cho et al. 2021 Cho et al. https://creativecommons.org/licenses/by/4.0/ This content is distributed under the terms of the Creative Commons Attribution 4.0 International license.

10.1128/mSystems.00943-21.2 FIG S2 RNA-seq and Ribo-seq validation. (A to D) Sequencing quality control. (A and B) Data reproducibility between RNA-seq duplicate data (A) and Ribo-seq duplicate data (B). (C and D) PC analysis of RNA-seq data (C) and Ribo-seq data (D). (E) Correlations between RNA-seq data-derived fold change and qPCR result-derived fold change data. (F) The qPCR fold change of photosynthesis-related genes under the HL and LT conditions compared to the CTRL condition. Abbreviations: ANT, antenna proteins; PSII, photosystem II; PSI, photosystem I; OCP, orange carotenoid protein; N, nitrogen assimilation. Download FIG S2, EPS file, 2.0 MB. Copyright © 2021 Cho et al. 2021 Cho et al. https://creativecommons.org/licenses/by/4.0/ This content is distributed under the terms of the Creative Commons Attribution 4.0 International license.

10.1128/mSystems.00943-21.6 TABLE S1 RNA-seq, Ribo-seq, and Term-seq statistics. Download Table S1, PDF file, 0.2 MB. Copyright © 2021 Cho et al. 2021 Cho et al. https://creativecommons.org/licenses/by/4.0/ This content is distributed under the terms of the Creative Commons Attribution 4.0 International license.

The DEGs from the two stress conditions were classified by their functions based on the Kyoto Encyclopedia of Genes and Genomes (KEGG) categories. The upregulated and downregulated DEGs under each condition annotated with KEGG Orthology (KO) were subjected to the KEGG enrichment analysis using KOBAS-i (Table 1) (33). The KEGG pathways with adjusted *P* values of less than 0.05 were classified as significantly altered pathways. Under the HL condition, the KEGG categories related to photosynthesis, such as “Photosynthesis,” “Photosynthesis – antenna proteins,” and “Porphyrin and chlorophyll metabolism,” were significantly downregulated. The first two pathways are under the higher hierarchy “Energy metabolism,” and the last one is under “Metabolism of cofactors and vitamins” (Fig. 1C). Such results reflect the decreased photosynthetic efficiency under HL. On the other hand, the downregulated DEGs under the LT condition had no KEGG enrichment regarding photosynthesis and only at the “Ribosome” metabolism (Table 1). The “Photosynthesis” category was instead upregulated, while only one significantly downregulated differentially expressed photosynthetic gene was found. While the “Energy metabolism” seems to be altered by both HL and LT stress, the photosynthetic genes under each photosynthesis-detrimental condition responded differently.

**TABLE 1:** Enriched KEGG pathways under stress conditions

DEG	KEGG pathway	Identifier	Input no.	Background no.	Adjusted *P* value
HL_UP	Nitrogen metabolism	syz00910	5	17	0.02963
HL_DOWN	Photosynthesis - antenna proteins	syz00196	12	15	5.41E−10
	Photosynthesis	syz00195	16	63	7.53E−08
	Porphyrin and chlorophyll metabolism	syz00860	7	53	0.030925
LT_UP	Photosynthesis	syz00195	10	63	0.038696
	Nitrogen metabolism	syz00910	5	17	0.038696
	ABC transporters	syz02010	12	87	0.038696
LT_DOWN	Ribosome	syz03010	10	60	0.001284

Gene ontology (GO) enrichment analysis was conducted with the DEGs (see Fig. S3 at https://figshare.com/s/67a5c10e6c6aa310f667). The Venn diagram shows the number of DEGs that were commonly altered under both stress conditions as well as specific DEGS under each condition (Fig. 1D). As seen in the KEGG analysis, the photosynthetic genes were found to respond in different manners under HL and LT (Fig. 1C). Under the HL condition, “Photosynthesis (GO: 0015979)” and “Protein-chromophore linkage (GO: 0018298)” GO terms were enriched to be downregulated. The protein-chromophore linkage is a function related to the assembly of phycobiliprotein with its chromophore compartment, phycocyanobilin (PCB). The matured phycobilisome is assembled into an antenna form attached to photosystem II (34). This corresponds with previous studies reporting cellular responses of lowered phycobilisome assembly under excess illumination (35). However, the photosynthesis-related genes were found to be enriched in the LT-specific upregulated gene category. Among DEGs, 6 out of 12 genes downregulated under HL but upregulated under LT were photosystem I-related genes, which is inconsistent with the phenotypic data (Fig. 1A and B). Unlike for HL, the discordance between the transcriptome changes and the phenotypic changes under LT is shown through DEG functional analysis, and it can be suggested that the decrease in photosynthetic yield under LT may follow different inhibitory mechanisms beyond transcriptional changes. The transcriptome analysis data achieved from RNA-seq were further validated by quantitative PCR (qPCR). The genes under assay were selected primarily among photosynthesis-related genes. The comparison of the RNA-seq data-derived fold change and qPCR result-derived fold change showed *R*^2^ values of 0.92 (HL) and 0.96 (LT) (Fig. S2).

### Translatome changes under HL and LT conditions.

Due to the unexpected discrepancy between the photosynthetic yield and transcriptional levels of photosynthetic genes under LT conditions, we hypothesized that the related genes could be regulated at the posttranscriptional level. To this end, ribosome profiling enables the monitoring of protein synthesis efficiency at a genome-wide scale using the deep sequencing of ribosome-protected mRNA fragments (RPFs) (36). Changes in the ratio between RPFs and mRNA transcript levels can be used to identify translational regulation under the conditions of interest. Ribosome profiling generated more than 33.1 million sequencing reads with an average read length of 30 bp, indicating an at least 111× sequencing depth (Table S1). The number of RPF reads per gene was then normalized using DEseq2 (see Table S3 at https://figshare.com/s/49e6c440505b0957b50d), resulting in a high degree of correlation between the biological duplicates (Pearson’s *r* > 0.97) and distinguished translational changes under each condition. In addition, we examined the overall translation efficiency (TE) by calculating the translation-to-transcription ratio for 3,251 coding sequences (CDSs). The average TE under the HL condition was higher than that under the CTRL condition (*R*^2^ = 0.89). In contrast, the average TE under the LT condition was the lowest among the three conditions (*R*^2^ = 0.76). Among the highly expressed genes, the TEs of photosynthesis-mediating genes, including *psaA*, *psaB*, *psbA3*, and *psbX*, were found to be decreased under the LT condition.

**FIG 2:**
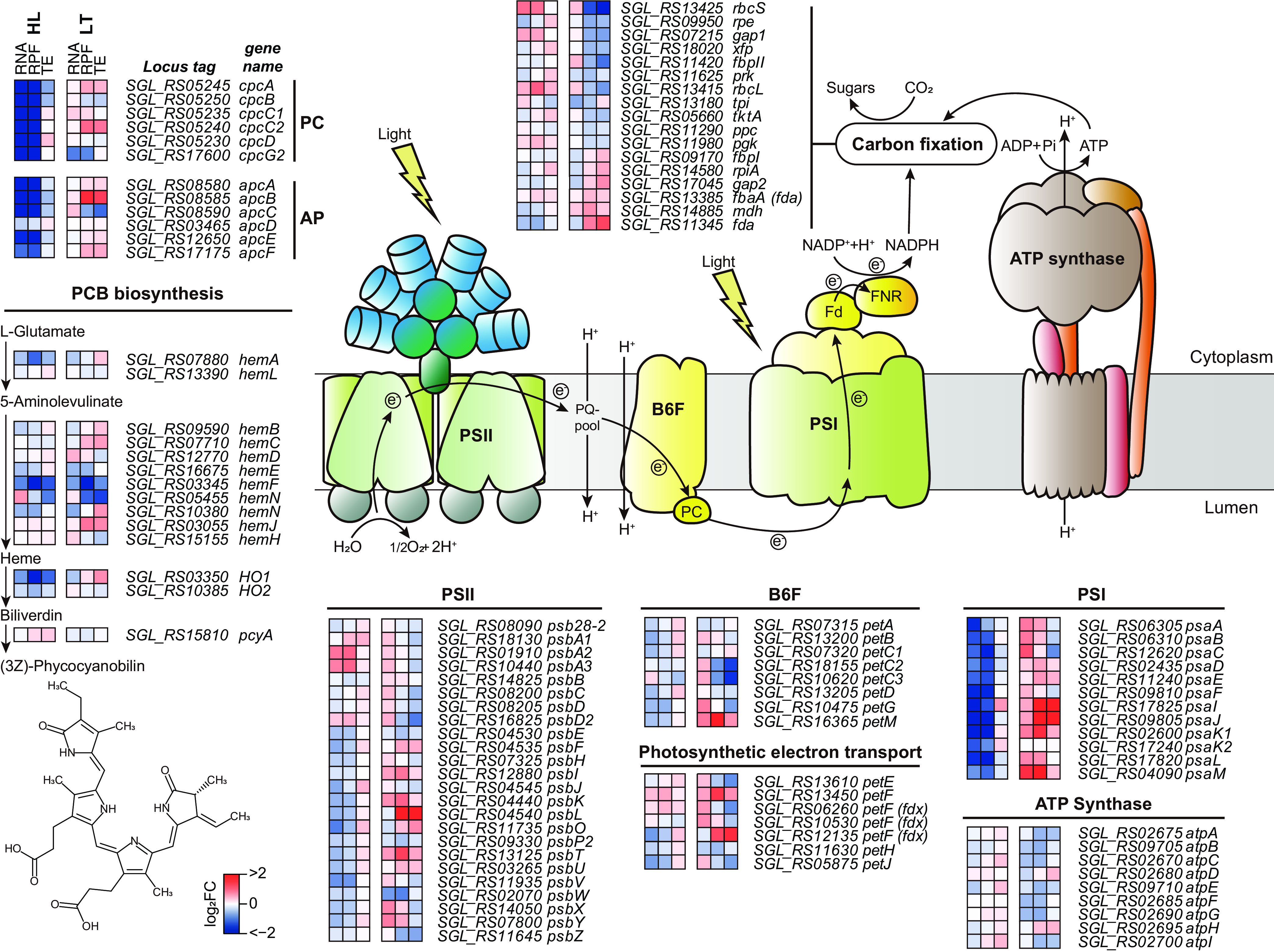
Expression patterns of photosynthetic machinery. Shown is a scheme of phycocyanobilin biosynthesis and photosynthetic electron flows that lead to carbon fixation. RNA and RPF expressions of the genes related to the photosynthetic machinery or pathway are provided as a heat map. Abbreviations: RPF, ribosome-protected fragment; PS, photosystem; LHC: light harvesting complex; OEC: oxygen evolving complex; PQ, plastoquinone; B6F, cytochrome *b*_6f_ complex; PC, plastocyanin; Fd, ferredoxin; FNR, ferredoxin-NADP^+^ reductase; PCB, phycocyanobilin.

For further analysis, we investigated the transcription, translation, and TE of the photosynthesis and carbon fixation-related genes based on KEGG categorization (Table S4). In addition, phycobiliproteins, which are light-harvesting antenna complexes of PSII, such as phycocyanin, phycoerythrocyanin, and allophycocyanin, were examined together (Fig. 2). Photosynthesis machinery is composed of antenna proteins, PSII, cytochrome *b*_6f_ complex, PSI, ATP synthase, and carbon fixation pathways. Under the HL condition, the photosynthesis-related genes, including the genes encoding the subunits of PSII, cytochrome *b*_6f_, PSI, and ATP synthase in the thylakoid membrane, were mostly downregulated at both the transcriptional and translational levels (Fig. S4). One notable exception was the D1/D2 reaction center complex in PSII, where D1, encoded by the *psbA* gene, was subjected to rapid turnover repair under HL stress, and the expression levels of D2, encoded by *psbD*, remained relatively constant (Fig. 2). In particular, the expression of antenna and PSI complex genes was downregulated in terms of both transcriptional and translational levels.

10.1128/mSystems.00943-21.3 FIG S4 RNA and RPF expression patterns. (A to C) Expression fold change of photosynthetic machinery: RNA fold change (A), RPF fold change (B), and Torso RPF fold change (C) under the HL and LT conditions compared to the control condition. (D and E) RNA expression patterns in gene extremities: RNA-seq profiles near the start codons (D) and the stop codons (E). Abbreviations: RPF, ribosome-protected fragment; ANT, antenna proteins; b6F, cytochrome *b*_6f_ complex; ATP, ATP synthase; C, carbon fixation; Chl, chlorophyll biosynthetic genes. Download FIG S4, EPS file, 2.9 MB. Copyright © 2021 Cho et al. 2021 Cho et al. https://creativecommons.org/licenses/by/4.0/ This content is distributed under the terms of the Creative Commons Attribution 4.0 International license.

10.1128/mSystems.00943-21.7 TABLE S4 List of genes related to photosynthesis, cyclic electron flow, and nitrogen assimilation. Download Table S4, PDF file, 0.2 MB. Copyright © 2021 Cho et al. 2021 Cho et al. https://creativecommons.org/licenses/by/4.0/ This content is distributed under the terms of the Creative Commons Attribution 4.0 International license.

Although LT stress decreased overall TE across the photosynthetic machinery, it did not severely affect specific photosynthetic machinery (Fig. S4). For example, the overall TE of PSII did not markedly decrease; however, the photosynthetic reaction center protein D1/D2, encoded by *psbAD*, and light-harvesting protein CP43/CP47, encoded by *psbBC*, showed low TEs (Fig. 2). In addition, while the transcriptional and translational levels of PSI under the LT condition increased, the TE of the photosynthetic reaction center protein family, *psaAB*, decreased. Other photosynthetic machinery components, such as the cytochrome *b*_6f_ complex and ATP synthase, were downregulated at the transcriptional and translational levels. The carbon fixation pathway under the LT condition was also downregulated at the translational level, while the transcriptional level was maintained. The translation of genes involved in the carbon fixation metabolism, including *rbcL*, *rbcS*, *pgk*, and *gap1*, was downregulated, resulting in decreased TE.

Taken together, the transcriptome and translatome analyses showed that the photosynthetic response and photoinhibition mechanism by HL and LT stresses have different modes. HL stress induces intensive downregulation in both the transcriptional and translational levels of specific photosynthetic machinery components such as phycobiliproteins and PSI. However, LT stress affects the TE of the photosynthesis-related genes, causing the inhibition of photosynthesis and carbon fixation.

### Translation buffering under LT stress.

**FIG 3:**
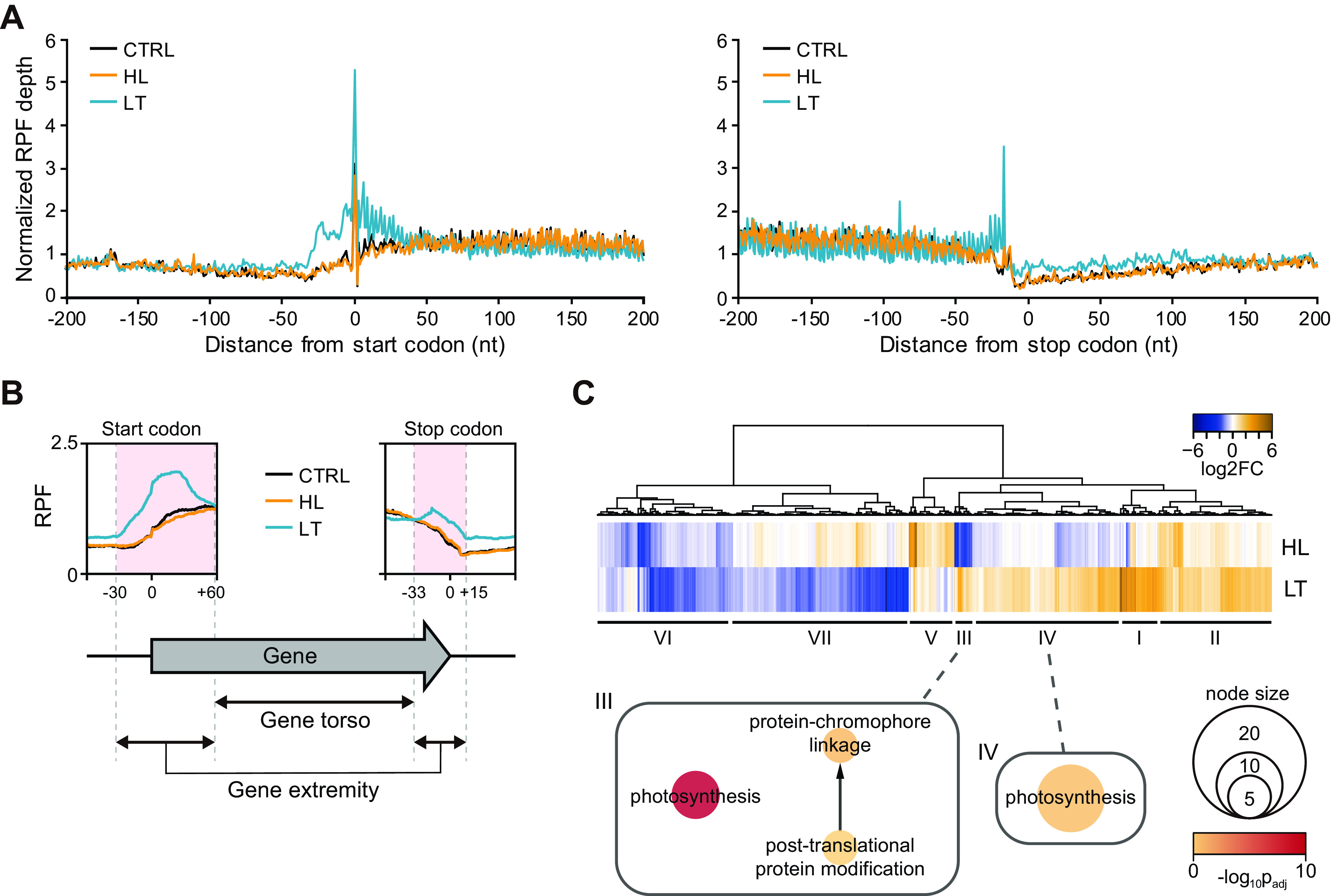
RPF abundance in gene extremities. (A) RPF depths under CTRL, HL, and LT conditions near the start (left) and stop (right) codons of all CDSs in the *Synechocystis* 6803 genome are shown. (B) The gene extremity and torso were distinguished based on the average RPF profile. (C) The genes with significantly altered RPF depths at gene extremities were clustered by the FC at the gene extremity under the HL and LT conditions compared to the CTRL condition. GO enrichment networks are presented in a circular force directed layout. Abbreviations: CDS, coding sequence; FC, fold change.

We investigated how the translation and TE decreased under the LT condition compared to other conditions. First, we examined the ribosome-binding profiles between −200 and +200 bp from the start and stop codons for all CDSs (Fig. 3A and B). Interestingly, the ribosomes were enriched near the start codon and stop codon under the LT condition compared to the HL and CTRL conditions (Fig. 3B), although their transcriptome changes were almost similar in all three conditions (Fig. S4D and E). These observations indicate possible ribosome stalling at the approximates of the UTRs under the LT condition.

To examine which genes were significantly affected by ribosome stalling at the start and stop codon approximates, the CDSs and their approximate regions were separated into two groups according to the RPF level of the LT condition compared to other conditions; the gene extremity regions included −30 to +60 bp from the start codon and −33 to +15 bp from the stop codon, and the gene torso region included the remaining internal CDSs, i.e., +61 from the start codon −33 from the stop codon (Fig. 3B). The changes in the RPF of each region were calculated compared to the CTRL condition. Then, the RPF changes in the extremity region were grouped into seven clusters through hierarchical clustering (Fig. 3C). Among them, clusters III and IV showed increased RPF changes in the extremity region. GO enrichment analysis of the genes grouped in these clusters revealed that the photosynthesis-related GO terms, “Photosynthesis (GO: 0015979)” and “Protein-chromophore linkage (GO: 0018298),” were enriched in clusters III and IV, suggesting that ribosome stalling at the gene extremity under the LT condition could be related to photosynthesis-related genes in the LT-specific mode.

### Determination of TU architecture by integrating 5′- and 3′-end information.

**FIG 4:**
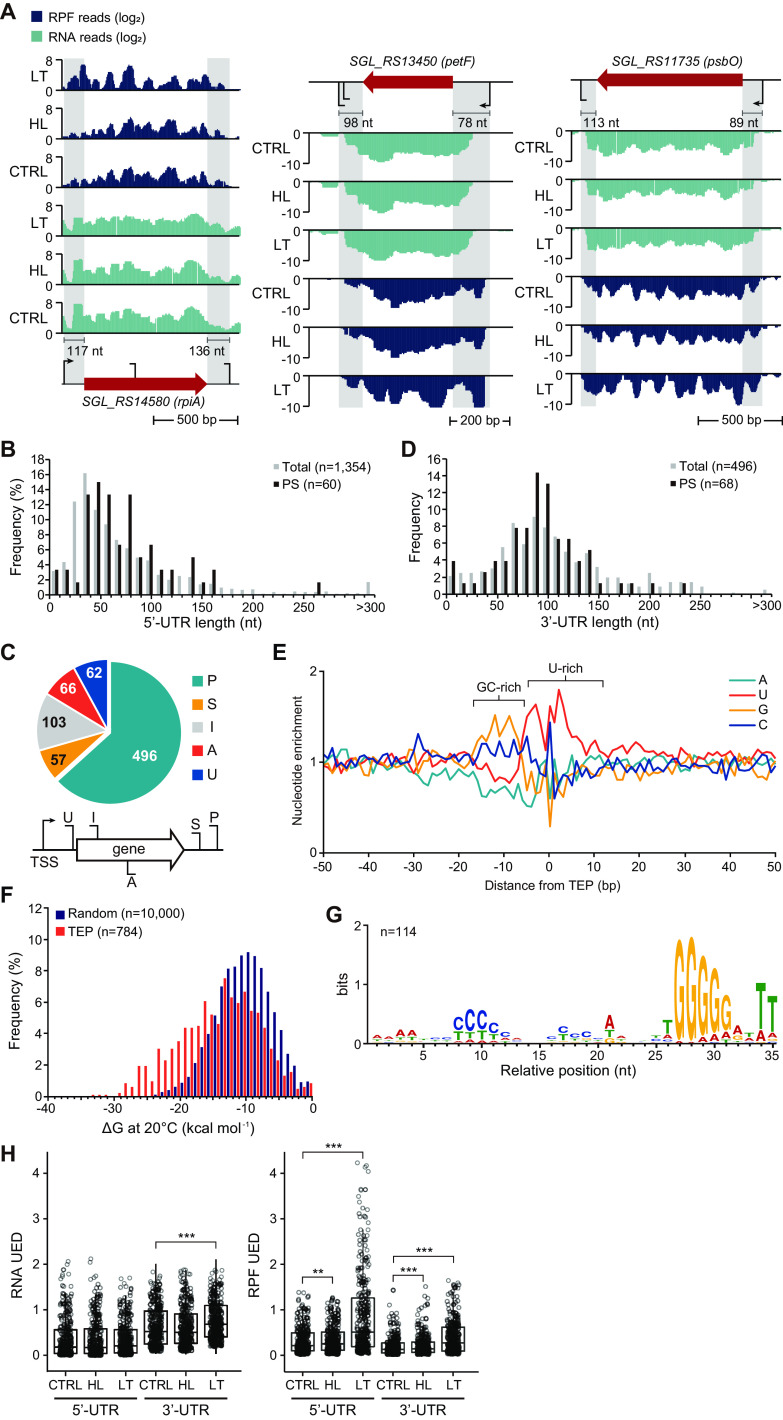
RNA and RPF profiles at 5′-UTR and 3′-UTR of the genes. (A) Examples of the genes showing RPF accumulation at their 5′-UTRs: SGL_RS14580 (*rpiA*, ribose-5-phosphate isomerase), SGL_RS13450 (*petF*, ferredoxin-1), and SGL_RS11735 (*psbO*, photosystem II manganese-stabilizing polypeptide). (B) 5′-UTR length distributions of total genes and photosynthesis-related genes. (C) The identified TEPs were categorized by comparing their positions with annotated genes. Abbreviations: P, primary; S, secondary; I, intragenic; A, antisense; U, upstream. (D) 3′-UTR length distributions of total genes and photosynthesis-related genes. (E) Nucleotide enrichment analysis of positions from −50 to +50 relative to TEP. (F) Free energy value distribution of sequences from −40 to TEP compared to 10,000 random intergenic sequences. (G) Motif searching upstream of TEP was performed using MEME. (H) The 5′-UED and 3′-UED were calculated for RNA or RPF depth by dividing the RNA or RPF depth at UTR with the RNA or RPF depth at whole TU, respectively. *, *P* value < 0.05; **, *P* value < 0.01; ***, *P* value < 0.001. Abbreviations: TU, transcription unit; TEP, 3′-end positions; UTR, untranslated region, UED, UTR enrichment degree.

Although the LT-specific accumulation of ribosomes near both ends of the CDS was observed, ribosome enrichment was also identified beyond the gene extremity in the UTR where translational regulation occurs, as shown in *rpiA*, encoding a protein involved in carbon fixation, *petF*, encoding a cytochrome *b*_6f_ subunit, and *psbO*, encoding a PSII subunit (Fig. 4A). For the systematic analysis of this phenomenon, RPF patterns at the 5′- and 3′-UTRs were examined. The 5′-UTR information of each CDS was obtained from the transcription start site (TSS) determined by dRNA-seq (19, 20). A total of 1,354 5′-UTRs with lengths ranging from 0 to 1,909 nucleotides (nt) were detected. The median length of all identified 5′-UTRs was 53 nt, while the photosynthesis-associated genes showed a longer median length, 60 nt (Fig. 4B).

For the determination of 3′-UTR, we identified 784 transcript 3′-end positions (TEPs) using Term-seq (31). The TEPs were classified into five categories according to their location relative to the CDS: primary (P), secondary (S), intragenic (I), antisense (A), and upstream (U) (Fig. 4C and Table S5). When multiple TEPs downstream of the stop codon were assigned to a gene, the 3′-UTR was defined with the P-TEP. The 3′-UTR lengths were between 1 and 497 nt, and the median length was 92 nt. Unlike 5′-UTRs, the 3′-UTR length distribution of photosynthesis-associated genes had no significant length difference from that of total genes (Fig. 4D). We investigated the nucleotide composition near the TEPs, revealing that their characteristics were similar to those of canonical intrinsic terminators, with a GC-rich stable stem followed by a U-rich tract found in other bacteria, such as Escherichia coli and Bacillus subtilis (31, 37) (Fig. 4E). There are currently two known bacterial transcription termination mechanisms: Rho-dependent and -independent termination. When an organism utilizes both termination strategies, the free energy distribution of the TEP approximates follows a bimodal distribution (38, 39). However, *Synechocystis* species have no Rho factors involved in transcription termination (20). Therefore, transcription termination is most likely mediated by the structure of the intrinsic terminator. This is supported by the single modal distribution of free energy at the TEP approximates (Fig. 4F). Conserved motif analysis of the 3′-UTR with 40-nt upstream regions from TEPs also revealed sequence characteristics of the canonical intrinsic terminator (Fig. 4E and G). Considering the similarity of the enriched motifs with other bacterial species, such as E. coli and B. subtilis, intrinsic transcription termination is the major mechanism.

10.1128/mSystems.00943-21.8 TABLE S5 List of transcript 3′-end positions (TEPs). Download Table S5, XLSX file, 0.04 MB. Copyright © 2021 Cho et al. 2021 Cho et al. https://creativecommons.org/licenses/by/4.0/ This content is distributed under the terms of the Creative Commons Attribution 4.0 International license.

Next, the TU was annotated to the genes whose TSS and P-TEP were both identified (Table S6). A total of 315 TUs were subjected to transcriptome and translatome analyses at the 5′-UTR and 3′-UTR. The RNA and RPF depths were calculated at the full length of the TU, and RNA and RPF depths at the 5′-UTR or the 3′-UTR were normalized by the depths across the full TU region, resulting in the UTR enrichment degree (UED). The RNA 5′-UEDs under the CTRL, HL, and LT conditions were similar regardless of the conditions, while the RPF 5′-UEDs were increased only under the LT condition (Fig. 4H). The RNA 3′-UED and RFP 3′-UED were both increased under the LT condition. The results support that LT stress causes significant ribosome enrichment at the 5′-UTR.

10.1128/mSystems.00943-21.9 TABLE S6 List of transcription units (TUs). Download Table S6, XLSX file, 0.02 MB. Copyright © 2021 Cho et al. 2021 Cho et al. https://creativecommons.org/licenses/by/4.0/ This content is distributed under the terms of the Creative Commons Attribution 4.0 International license.

### Ribosome stalling at the 5′-UTRs of photosynthesis-associated genes.

**FIG 5:**
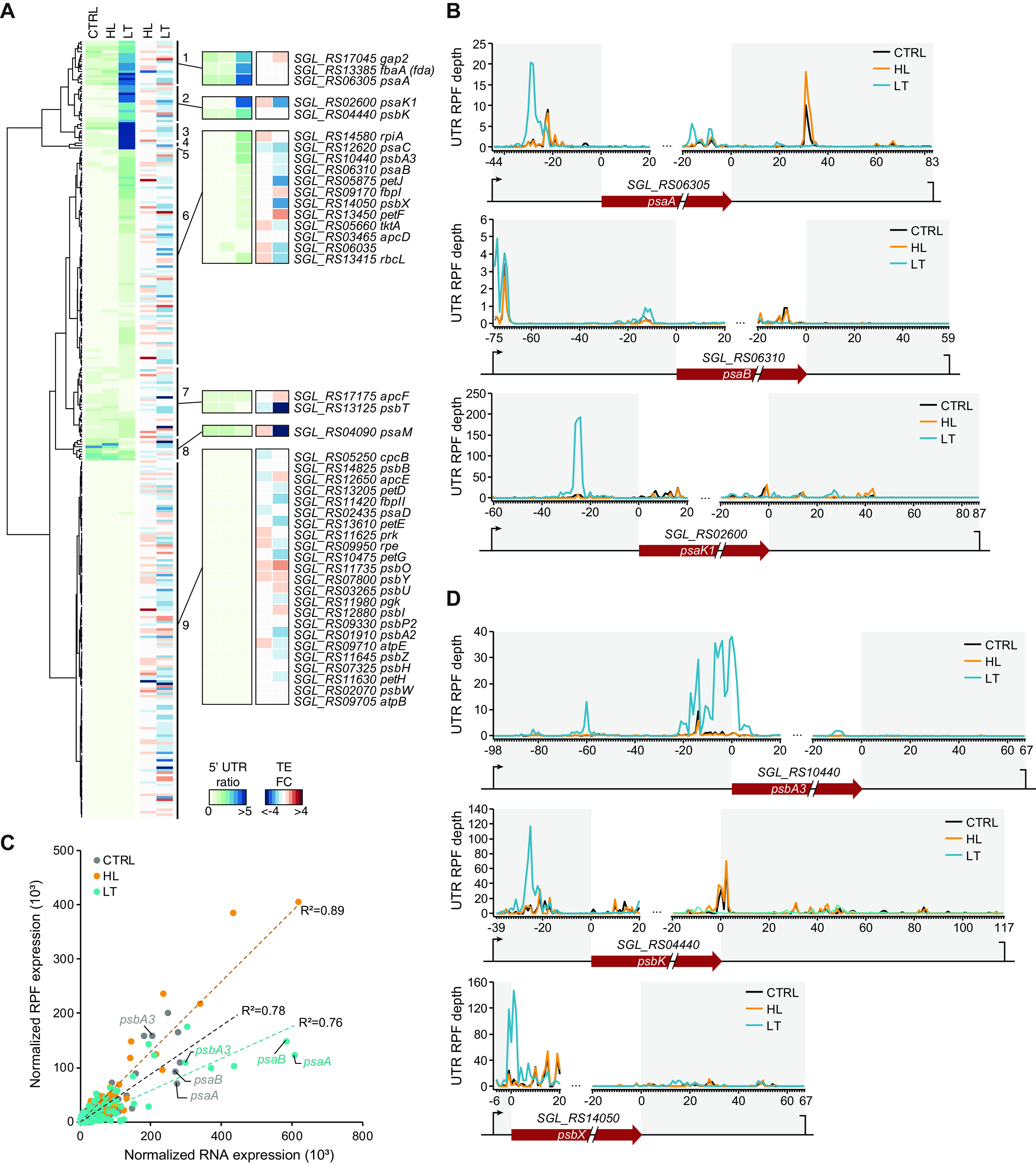
Accumulation of RPF at 5′-UTR and the translation efficiency of photosynthesis-related genes. (A) RPF 5′-UED of photosynthesis-related genes shown as a heat map. The gene clusters were generated by hierarchical clustering. (B) The expression profiles of photosystem I genes, of which RPF 5′-UED increased under the LT condition compared to the control condition. (C) The RNA and RPF expressions of each gene under CTRL, HL, and LT conditions were compared. (D) The expression profiles of PSII genes, of which RPF 5′-UED were increased under the LT condition compared to the CTRL condition.

The genes were clustered according to their RPF 5′-UED or 3′-UED patterns across the three conditions to analyze the biological functions affected by the ribosome enrichment at both of the UTRs. As a result of RPF 5′-UED clustering, most genes were clustered under either LT-specific UED-increased clusters (clusters 1 to 6) or nonchanging clusters (cluster 9) (Fig. 5A). In contrast, in the RPF 3′-UED clustering, more than half of the genes (71.4%) were included in the clusters whose RPF 3′-UED did not change across conditions (Fig. S5). Among 43 photosynthesis-associated genes, 17 were included in the cluster where the RPF 5′-UED was increased, and 11 were included in the cluster where the RPF 3′-UED was increased (Fig. 5A). Notably, more than half of the genes related to PSI, cytochrome *b*_6f_, and PSII had an increased RPF 5′-UED. For PSI, the RPF 5′-UED of *psaK1* (SGL_RS02600), *psaA* (SGL_RS06305), *psaB* (SGL_RS06310), and *psaC* (SGL_RS12620) had increased RPF 5′-UED (Fig. 5B). PsaK1 is thought to be the last assembled subunit in the PSI complex assembly process, although its function is currently unknown (40). PsaK1 has a 34% BLASTP identity to PsaG of Arabidopsis thaliana, which is absent in cyanobacteria. PsaG is involved in PSI core and antenna stabilization under low-light conditions (40, 41). In the case of *psaK1*, the RNA expression under the LT condition increased more than 2-fold compared to that under the CTRL condition (DESeq2 *P* value = 3.0 × 10^−12^) and increased more than 3-fold when the RPF expression of the whole length of the CDS was calculated (DESeq2 *P* value = 5.2 × 10^−36^). However, when the RPF of *psaK1* was recalculated by excluding the ribosomes accumulated in the extremities, the RPF expression was reduced 2-fold (DESeq2 *P* value = 5.2 × 10^−36^). The discrepancy between RNA and RPF expression levels indicates that although the transcriptional level significantly increased, the TE eventually decreased due to poor translation initiation or elongation rate. Assuming that PsaK1 has a function similar to that of PsaG, downregulation of *psaK1* under LT conditions could result in decreased PSI core stability. Indeed, *psaA* and *psaB* genes, which encode PSI core proteins, also showed increased RPF 5′-UED under LT conditions (Fig. 5C). The RNA and RPF expression of both *psaA* and *psaB* increased, but a translation buffering phenomenon was observed in which the increased level of translation was insufficient compared to the increased levels of transcripts (42, 43) (Fig. 5D). For PSII, the RPF 5′-UED of the PSII core D1 protein-encoding gene, *psbA3* (SGL_RS10440), and of *psbK* (SGL_RS04440), and *psbX* (SGL_RS14050), encoding the PSII reaction center K and X proteins, respectively, increased (Fig. 5D). Translation buffering was also observed in these genes, especially *psbA3* and *psbX*, with >2- and 3-fold decreases in TE compared to the case under the CTRL condition, respectively.

10.1128/mSystems.00943-21.4 FIG S5 UTR enrichment degrees (UEDs) of photosynthesis genes. (A) Clustering analysis of RPF 3′-UED. The genes related to photosynthesis are indicated separately (right). (B) Photosynthesis-related genes with increased RPF UEDs. Red arrows indicate increased UEDs under the LT condition compared to the control condition. *SGL_RS04440*, *SGL_RS09330*, *SGL_RS10440*, *SGL_RS11735*, *SGL_RS12880*, and *SGL_RS14050* are PSII genes, *SGL_RS06035*, *SGL_RS11630*, *SGL_RS13205*, *SGL_RS13450*, and *SGL_RS13610* are genes related to the cytochrome *b*_6f_ complex, *SGL_RS02600*, *SGL_RS06305*, *SGL_RS06310*, and *SGL_RS12620* are PSI genes, and *SGL_RS05660*, *SGL_RS09170*, *SGL_RS11980*, *SGL_RS13385*, *SGL_RS13415*, *SGL_RS14580*, and *SGL_RS17045* are genes related to carbon fixation. Abbreviation: ctrl, control. Download FIG S5, PDF file, 2.6 MB. Copyright © 2021 Cho et al. 2021 Cho et al. https://creativecommons.org/licenses/by/4.0/ This content is distributed under the terms of the Creative Commons Attribution 4.0 International license.

Taken together, the findings show that ribosome accumulation occurred globally at UTRs under the LT condition. In particular, ribosome stalling was detected in the 5′-UTR of photosynthesis-associated genes, resulting in the poor initiation of leading ribosomes (44). Therefore, the accumulation of ribosomes at the 5′-UTR can reduce TE under LT stress conditions.

## DISCUSSION

**FIG 6:**
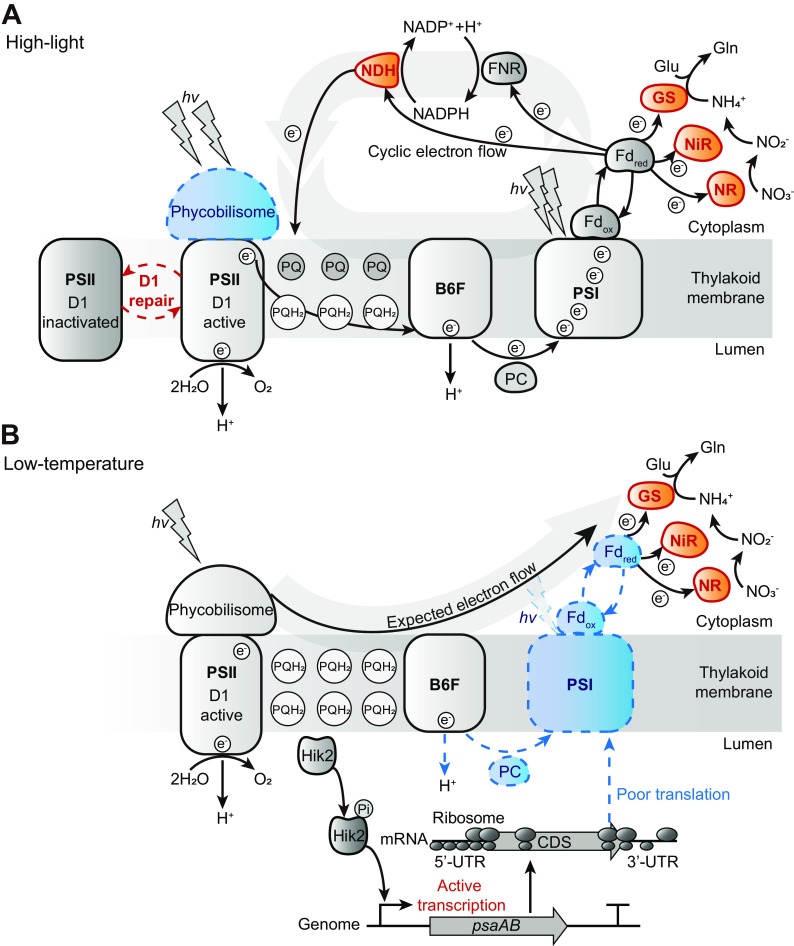
Photosynthetic machinery in response to HL and LT stresses. Proposed model showing the response of photosynthetic machinery from the perspective of the electron flow under HL (A) and LT (B) stresses. The red color indicates upregulation or activation of a certain gene or cellular function, and the blue indicates downregulation or deactivation. Abbreviations: PSII, photosystem II; PSI, photosystem I; B6F, cytochrome b6f; PQ, plastoquinone; PQH_2_, plastoquinol; Fd_ox_, oxidized ferredoxin; Fd_red_, reduced ferredoxin; NR, nitrogen reductase; NiR, ferredoxin-nitrite reductase; GS, glutamine synthetase; Glu, glutamate; Gln, glutamine; PC, plastocyanin; NDH, NADPH dehydrogenase; CDS, coding sequence; UTR, untranslated region.

In this study, the cellular transcriptome and translatome changes of *Synechocystis* sp. PCC 6803 were investigated to understand the molecular feedback of photosynthesis in response to HL and LT stresses using integrative multiomics analysis of RNA-seq, ribosome profiling, dRNA-seq, and Term-seq (31, 36). Under HL stress, the first response is to decrease the electron transfer rate by downregulation of the phycobiliprotein antenna protein attached to PSII (Fig. 6A). Except for the D1 protein with the repair system and D2 protein of PSII, most genes encoding PSII, cytochrome *b*_6f_, and ATP synthase were downregulated in terms of both transcriptional and translational levels. In particular, HL stress led to irreversible downregulation of PSI. To prevent photodamage in PSI, the electron efflux was accelerated by increasing the cyclic electron flow and reducing power-demanding processes such as nitrogen assimilation utilizing glutamine synthetase, ferredoxin-nitrite reductase, and ferredoxin-nitrate reductase (ferredoxin-NR) (Fig. 6A and Table S4).

LT stress causes a decline in membrane fluidity, where the photosynthesis machinery is located (45). Diminished membrane fluidity can attenuate the migration of electrons through the PQ/PQH_2_ pool in the thylakoid membrane and induce an imbalance of redox potential (13) (Fig. 6B). In response to the high reduction state of the PQ/PQH_2_ pool, chloroplast sensor kinase (CSK) is autophosphorylated and induces the transcription of *psaA* and *psaB*. A recent study found evidence that *Synechocystis* Hik2 is a homolog of CSK responsible for sensing the redox state of the PQ/PQH_2_ pool (46). The Fe-S cluster of the Hik2 has the conformation of [3Fe-4S], which affects the protein structure when the redox potential changes. Thus, it can be hypothesized that the increased RNA expression level of PSI only under the LT condition could be a result of the Hik2 activity, although the expression level of Hik2 did not significantly alter (Fig. 6B). However, further biochemical studies are required to elucidate the mechanism of Hik2 under stress conditions. The expression of the nitrogen assimilation pathway was also highly increased, possibly creating momentum for the electron flow (Fig. 6B and Table S7). The LT stress also triggered a decrease in TE in the core genes of PSII, cytochrome *b*_6f_, PSI, and ATP synthase in the thylakoid membrane, and plastocyanin in the electron transport chain, even though the transcription of the genes was highly upregulated. Interestingly, the ribosomes were highly positioned at the extremity of the CDS of the PSI genes, not at the torso portion. A similar ribosomal accumulation in the 5′-UTR was frequently found not only in PSI but also among other photosynthesis-associated genes. The longer 5′-UTR length distribution in photosynthesis-associated genes may affect the secondary-structure formation of the transcripts and further stabilize them under LT conditions. The increased ribosome occupancy at the 5′-UTR and decreased TE are termed translational buffering. In addition to membrane integrity, temperature also affects cellular biochemistry, such as pH, CO_2_ and O_2_ solubility, and enzyme activity (13). Furthermore, unlike with the HL condition, where the TE slightly increases, the translation rate decreases substantially under LT conditions (47, 48). In the current study, the RPF profiles under the LT condition showed a substantial increase only near the start and stop codons compared to the CTRL and HL conditions. The ribosomal proteins were downregulated at the transcriptional level, and the antisense rRNA level was increased. Among the downregulated ribosomal proteins under LT conditions, 50S ribosomal proteins, L5 and L20, are involved in the structural formation of the ribosomal complex (49, 50). This suggests that the formation of the ribosomal complex is affected by LT. Furthermore, the elongation factor, Ts, and translation initiation factor, Sui1, were downregulated, which involved translation initiation and elongation. Collectively, these results suggest that the reduced efficiency of translation initiation and elongation leads to the enrichment of RPFs at the 5′-UTR.

10.1128/mSystems.00943-21.10 TABLE S7 Primers used for quantitative real-time PCR. Download Table S7, PDF file, 0.09 MB. Copyright © 2021 Cho et al. 2021 Cho et al. https://creativecommons.org/licenses/by/4.0/ This content is distributed under the terms of the Creative Commons Attribution 4.0 International license.

Under stress conditions, bacteria can utilize an RNA degradation mechanism to regulate their physiology (51). In response, the ribosome docking can protect the transcripts from degradation catalyzed by the RNA degradosome. The RNA degradation mechanism in *Synechocystis* sp. PCC 6803 was investigated to check whether ribosome enrichment in the UTR is related to the mechanism. While the RNA degradosome is not fully identified in *Synechocystis*, it could be examined based on other model bacterial species, such as Escherichia coli and Bacillus subtilis (52–54). The RNA degradosome composing gene list was identified, including the core RNA degradosome proteins and the associated proteins (Fig. S6). Under both stress conditions, the genes coding the core RNA degradosome did not show significant expression upregulation at the transcription level or translation level. Instead, the overall expression was decreased under both conditions, where the significantly downregulated genes under the LT condition were RNase E at the transcription level (log_2_ FC = −1.45) and RNase J, PNPase, and CrhR at the translation level (log_2_ FC = −1.79, −1.28, and −1.01, respectively). Based on the expression levels of the core degradosome, the RNA degradation activity may be lower under the LT condition, and the ribosome enrichment under LT is unlikely to be caused by RNA degradation.

10.1128/mSystems.00943-21.5 FIG S6 Expression levels of RNA degradosome-related genes. The genes related to the RNA degradation mechanism in *Synechocystis* sp. PCC 6803 were examined. Download FIG S6, EPS file, 2.0 MB. Copyright © 2021 Cho et al. 2021 Cho et al. https://creativecommons.org/licenses/by/4.0/ This content is distributed under the terms of the Creative Commons Attribution 4.0 International license.

Recent advances in multiomic technology have facilitated quantitative tracking of changes in the genome, transcriptome, and translatome, which enable the analysis of various regulations that orchestrate the cellular system by integrating disparate data. In this context, this work analyzed the incoherence between the phenotypic measurements of the cellular responses to stress conditions using the integrative analysis of multiomics data. Along with the transcription unit architecture constructed by transcription start sites and transcript 3′-end positions, we showed that high-light stress induced a photodamage response, and low-temperature stress impaired the translation efficiency of photosynthesis-associated genes. Based on the fact that the cyanobacterial engineering requires understanding of photosynthetic efficiency under diverse environmental conditions such as high-light and low-temperature stresses, this study provides pivotal information for engineering cyanobacterial cell factories.

## MATERIALS AND METHODS

### Strains and culture conditions.

Glucose-tolerant *Synechocystis* sp. PCC 6803 cells were grown at 30°C under continuous illumination from fluorescent lamps at 50 μmol photons m^−2^ s^−1^ in BG-11 medium with aeration in 1% CO_2_ balanced air at a flow rate of 0.1 vol vol^−1 ^min^−1^. Cells in the early exponential phase of growth (optical density at 730 nm = 0.3) were transferred to stress conditions of HL (400 μmol photons m^−2^ s^−1^) and LT (20°C). After 1 h, cultures were sampled for RNA-seq and Term-seq or treated with chloramphenicol (Sigma-Aldrich, MO) for 5 min and sampled for ribosome profiling (Ribo-seq).

### Photosynthetic activity measurements.

To determine the photosynthetic activity of *Synechocystis* sp. PCC 6803, 400 ml of precultivated *Synechocystis* sp. PCC 6803 cultures in the early exponential phase of growth (optical density at 730 nm = 0.3) were transferred to the control condition (30°C) and the low-temperature condition (20°C). Cultures were then incubated for an hour under each condition. Before measuring quantum yield and electron flux with MINI-PAM-II (WALZ, Germany), the cultures were incubated without light for 15 min. To ensure a constant sample temperature throughout the measurement, a temperature control accessory was installed to the MINI-PAM-II. The experiment was conducted in a triplicate manner.

### RNA-seq library construction.

One hundred milliliters of culture under each stress condition was harvested by centrifugation at 4,000 × *g* and 4°C for 5 min. The pellets were immediately resuspended in 300 μl of lysis buffer composed of 10 mM Tris-HCl (pH 7.6), 5 mM MgCl_2_, and 40 mM NaCl. The resuspended cells were then frozen using liquid nitrogen and ground to a fine powder using a pestle and mortar. The powdered cells were immediately transferred to 700 μl of QIAzol lysis reagent (Qiagen, CA) for stabilization, briefly vortexed, and incubated at room temperature for 5 min. To isolate the RNA, 140 μl of chloroform (Sigma-Aldrich) was added, vortexed for 15 s, and incubated at room temperature for 3 min. The aqueous phase was isolated by centrifugation at 4°C for 15 min at 15,000 × *g* and mixed with 1.5 times the volume of absolute ethanol. Total RNA was purified using the miRNeasy minikit (Qiagen) according to the manufacturer’s instructions. The rRNA from the total RNA was removed using the Ribo-Zero rRNA removal kit for bacteria (Illumina, CA) according to the manufacturer’s instructions. The RNA-seq library was constructed as previously described (55). As the rRNA-depleted total RNA does not require a purification step for poly(A)-tailed mRNA, the RNA-seq library was prepared from the RNA fragmentation step of the TruSeq Stranded mRNA library prep kit (Illumina).

### Ribo-seq library construction.

The collected cells after chloramphenicol treatment were immediately resuspended in 300* μl* of lysis buffer composed of 10 mM Tris-HCl (pH 7.6), 5 mM MgCl_2_, 40 mM NaCl, 1% Triton X-100, and 1% chloramphenicol at 34 mg ml^−1^. Resuspended cells were frozen using liquid nitrogen and ground to a fine powder using a pestle and mortar. The ground cells were centrifuged for 10 min at 20,000 × *g* and 4°C to recover the soluble supernatants. After lysate purification, the Ribo-seq library was constructed using the streamlined ribosome profiling protocol without the tRNA depletion step (56). The lysate containing 50 μg of total RNA was treated with 400 U of MNase (New England BioLabs, MA), 20 μl of 10× MNase buffer, and 2 μl of 100× bovine serum albumin (BSA) at 37°C for 2 h. The reaction was quenched by adding 2.5 μl of 500 mM EGTA. The samples were loaded onto illustra MicroSpin S-400 HR columns (GE Healthcare, IL), which had been washed with 500 μl of washing buffer (50 mM Tris-HCl [pH 8], 250 mM NaCl, 50 mM MgCl_2_, 25 mM EGTA, and 1% Triton X-100) three times. The column was centrifuged at 4°C for 2 min at 400 × *g*, and the flowthrough was further purified using phenol-chloroform-isoamyl alcohol (25:24:1; Thermo Fisher Scientific, MA) and ethanol precipitation. rRNA was removed using the Ribo-Zero rRNA removal kit. The RNA fragments between 26 and 32 nt were size selected by gel electrophoresis for 65 min at 200 V using a 15% polyacrylamide Tris-borate-EDTA (TBE)-urea gel (Invitrogen, CA). The RNAs in the excised gel were eluted in 400 μl of RNA gel extraction buffer (300 mM sodium acetate [pH 5.5], 1 mM EDTA, and 0.25% [wt vol^−1^] SDS) by freezing at −80°C for 30 min, followed by incubation at 37°C for 4 h with gentle mixing. The eluted RNAs were isolated by ethanol precipitation and purified again with an RNeasy MinElute column (Qiagen). The samples were denatured at 80°C for 90 s and then equilibrated to 37°C. For the dephosphorylation reaction, 10 U of T4 polynucleotide kinase (T4 PNK; New England BioLabs), 20 U of SUPERase-In, and 5 μl of 10× T4 PNK buffer were added to the sample and incubated at 37°C for 1 h. The dephosphorylated RNAs were purified using an RNeasy MinElute column. The sequencing library was constructed using the NEBNext multiplex small RNA library prep set for Illumina (New England BioLabs) according to the manufacturer’s instructions. The final library of 150 bp was size selected by gel electrophoresis for 90 min at 100 V using a 2% agarose gel dyed with SYBR gold nucleic acid gel stain (Bio-Rad, CA). After reverse transcription, the library was amplified using the CFX96TM real-time PCR detection system with observation until the reaction reached the semiplateau phase. The concentration of the final library was measured with a Qubit 2.0 fluorometer (Invitrogen), while the size distribution was assessed with an Agilent 2200 TapeStation system (Agilent, CA).

### Term-seq library construction.

To construct the library for 3′-end enriched sequencing, a total of at least 1 μg of rRNA-removed mRNA was prepared. The Term-seq library was constructed as previously described (31). Briefly, the prepared RNA was ligated to RNA 3′ adapters of the sequence 5′-NNAGATCGGAAGAGCGTCGTGT-3′ and chemically fragmented for 90 s at 72°C using RNA 10× fragmentation buffer (Thermo Fisher Scientific) and purified using 2.2× volume AMPure XP beads (Beckman Coulter, IN). The 3′ RNA adapter-ligated RNA was then reverse transcribed by a reverse transcription primer of the sequence 5′-TCTACACTCTTTCCCTACACGACGCTCTTC-3′, and a cDNA 3′ adapter of the sequence 5′-NNAGATCGGAAGAGCACACGTCTGAACTCCAGTCAC-3′ was ligated to the cDNA. The cDNA adapter ligated at both ends was then amplified using a CFX96TM real-time PCR detection system until reaction plateau with the following primer set: forward primer, 5′-AATGATACGGCGACCACCGAGATCTACACTCTTTCCCTACACGACGCTCTTCCGATCT-3′, and reverse primer, 5′-CAAGCAGAAGACGGCATACGAGAT-6 mer-index- GTGACTGGAGTTCAGACGTGTGCTCTTCCGATCT-3′.

### Data analysis.

The RNA-seq and Ribo-seq reads were trimmed by removing PhiX sequences and reads with a quality score of less than 0.05, and the remaining reads were then mapped to the *Synechocystis* sp. PCC 6803 genome (RefSeq assembly accession number GCF_000009725.1) using CLC Genomics Workbench (similarity fraction = 0.9; identity fraction = 0.9). The mapped reads were exported to Binary Alignment/Map files and converted to a general feature format for profile analysis and visualization. The raw read count for the *Synechocystis* sp. PCC 6803 genome was exported for normalization using DESeq2 (57). For the Ribo-seq data, sequences that were mapped to the tRNA and rRNA were removed to ensure that gene read count normalization was unaffected by the efficiency of tRNA of rRNA removal for each library. To assign the range of gene extremity and gene torso, the average RPF value for the CTRL and HL conditions at each relative position from the start and stop codon was calculated. The position where the RPFs of the LT condition decreased to the average RPF value of the CTRL and HL conditions was designated the boundary position separating the extremity and torso.

TSS information was pooled from two previous studies (22, 23). Term-seq analysis was performed based on a previously described method (31). After the barcode sequence and two random nucleotides ligated with the adapters were removed, the position of the 5′ end of each read was mapped to the genome in the reverse strand. Only the TEPs commonly detected in the replicates were used for further analysis. The P-TEPs were identified by obtaining the position with the highest coverage within 250 nt downstream of the stop codon. The remaining TEPs located within the same range as the P-TEPs were categorized as S-TEPs. The P- and S-TEPs were manually curated by comparing them with the RNA-seq profile. The TEPs in the I-, A-, and U-TEPs were identified manually, where the position with the highest coverage and correlation to the RNA-seq profile was identified. The Gibbs free energy for the secondary structure was calculated using ViennaRNA package 2.0 (58). Motif search was performed using MEME version 5.0.0, with the following parameters: maxsize of 1,000,000, minw of 10, maxw of 35, and mod of zoops. To calculate the UED, the depth of each TU was first calculated. From each TSS to TEP associated with each gene, the RNA or RPF depth of each position was added, and this was divided by the length of the TU. The 5′-UED was calculated by adding the RNA or RPF depth of each position from the TSS to the −1 position of the gene start codon, dividing it by the 5′-UTR length, and dividing by the depth of the TU:

(1) $${\text{R}}_{5\text{utr}}=\;\frac{\sum_{i=t}^{s-1}d_{i}/l_{5\text{utr}}}{\sum_{i=t}^{p}d_{i}/l_{\text{tu}}}$$

where R_5utr_ is the 5′-UED, *l*_5utr_ is the length of the 5′-UTR, *l*_tu_ is the length of the TU, *d_i_* is the RNA or RPF depth of each genomic position, *t* is the position of the TSS, *s* − 1 is the −1 position of the start codon, and *p* is the TEP. 3′-UED was calculated by adding the RNA or RPF depth of each position from the +1 position of the gene stop codon to the TEP, dividing it by the 3′-UTR length, and dividing by the depth of the TU:

(2) $${\text{R}}_{3\text{utr}}=\;\frac{\sum_{i=e+1}^{p}d_{i}/l_{3\text{utr}}}{\sum_{i=t}^{p}d_{i}/l_{\text{tu}}}$$

where R_3utr_ is the 3′-UED, *l*_3utr_ is the length of the 3′-UTR, *l*_tu_ is the length of the TU, *d_i_* is the RNA or RPF depth of each genomic position, *p* is TEP, *e *+ 1 is the +1 position of the stop codon, and *t* is the position of the TSS.

### DEG functional enrichment analysis.

The DEG functional enrichment analyses were conducted using the KEGG Orthology or GO terms (59, 60). The amino acid sequences obtained from the *Synechocystis* sp. PCC 6803 genome sequence (RefSeq assembly accession number GCF_000009725.1) were used for the KEGG ID and GO term annotations using EggNOG (61). The KEGG pathway enrichment of the upregulated DEGs and downregulated DEGs was calculated by using KEGG Orthology-Based Annotation System (KOBAS) 3.0 (also known as KOBAS-i) with options “Databases: KEGG PATHWAY; Statistical test method: hypergeometric test/Fisher’s exact test; FDR correction method: Benjamini and Hochberg” (33). The GO analyses of the DEGs of each condition were conducted by using BiNGO applications on the CytoScape (62). The options used for the analysis were “Hypergeometric test; Benjamini and Hochberg FDR correction.” The significance of the enrichment was determined by the adjusted *P* value, in which enrichments with an adjusted *P* value of less than 0.05 were classified as enriched.

### Quantitative real-time PCR.

First-strand cDNA was synthesized from 200 ng of rRNA depleted RNA by using the SuperScript III first-strand synthesis system (Invitrogen) following the manufacturer’s instructions. The amplification of the cDNAs was monitored on a StepOnePlus real-time PCR system (Thermo Fisher Scientific) with the KAPA SYBR FAST qPCR master mix kit (Thermo Fisher Scientific). The conditions used for amplification were as follows: 95°C for 3 min, 95°C for 10 s, 58°C for 20 s, and 72°C for 20 s for 40 cycles. The sequences of the primers used for amplification are indicated in Table S7.

### Data availability.

All of the sequencing raw reads, including RNA-seq, Ribo-seq, and Term-seq, for each condition can be found at the National Center for Biotechnology Information under BioProject number PRJNA666973.
