# Supplementary material for: Different Regulatory Modes of Synechocystis sp. PCC 6803 in Response to Photosynthesis Inhibitory Conditions
Source: mSystems. 2021 Dec 7;6(6):e00943-21. doi: 10.1128/mSystems.00943-21 (PMC8651088; doi:10.1128/mSystems.00943-21)
Supplement: TABLE S1 [file msystems.00943-21-st001.pdf]

Table S1. RNA-seq, Ribo-seq, and Term-seq statistics.

			**RNA-seq statistics**			
	**CTRL1**	**CTRL2**	**HL1**	**HL2**	**LT1**	**LT2**
Total reads	18,318,733	19,927,160	19,819,685	15,109,971	39,546,059	33,920,908
Uniquely mapped reads	16,837,898	18,716,303	15,500,272	13,061,881	33,429,851	28,732,828
Mapped read length in average	50.97	50.97	50.97	50.97	50.98	50.98
Coverage	217.44	241.69	200.16	168.68	431.78	371.12
			**Ribo-seq statistics**			
	**CTRL1**	**CTRL2**	**HL1**	**HL2**	**LT1**	**LT2**
Total reads	48,245,619	45,311,369	34,338,501	33,079,365	41,012,878	40,009,379
Uniquely mapped reads	23,165,654	20,947,496	15,957,645	14,881,321	30,990,052	27,216,504
Mapped read length in average	29.36	29.34	29.68	29.48	31.49	31.62
Coverage	172.32	155.71	112.00	111.15	247.24	218.03

	**Term-seq statistics**	
	**Pooled1**	**Pooled2**
Total reads	3,523,487	3,242,267
Uniquely mapped reads	2,750,566	2,556,730
Mapped read length in average	45.49	45.39
Coverage	31.70	29.40
