# Supplementary material for: Different Regulatory Modes of Synechocystis sp. PCC 6803 in Response to Photosynthesis Inhibitory Conditions
Source: mSystems. 2021 Dec 7;6(6):e00943-21. doi: 10.1128/mSystems.00943-21 (PMC8651088; doi:10.1128/mSystems.00943-21)
Supplement: TABLE S4 [file msystems.00943-21-st004.pdf]

Table S4. List of genes related to photosynthesis, cyclic electron flow and nitrogen assimilation.

Gene ID	Gene name	Function
		**Photosynthesis**
		Allophycocyanin (AP)
SGL_RS08580	*apcA*	allophycocyanin alpha chain
SGL_RS08585	*apcB*	allophycocyanin subunit beta
SGL_RS08590	*apcC*	photosystem I reaction center subunit XII
SGL_RS03465	*apcD*	allophycocyanin subunit alpha-B
SGL_RS12650	*apcE*	phycobiliprotein ApcE
SGL_RS17175	*apcF*	allophycocyanin subunit beta-18
		Phycocyanin (PC)
SGL_RS05245	*cpcA*	C-phycocyanin alpha chain
SGL_RS05250	*cpcB*	C-phycocyanin beta chain
SGL_RS05235	*cpcC1*	photosystem I reaction center subunit XII
SGL_RS05240	*cpcC2*	photosystem I reaction center subunit XII
SGL_RS05230	*cpcD*	photosystem I reaction center subunit XII
SGL_RS17600	*cpcG2*	phycobilisome rod-core linker polypeptide CpcG
		PSI
SGL_RS06305	*psaA*	photosystem I core protein PsaA
SGL_RS06310	*psaB*	photosystem I P700 chlorophyll a apoprotein A2
SGL_RS12620	*psaC*	photosystem I iron-sulfur center
SGL_RS02435	*psaD*	photosystem I reaction center subunit II
SGL_RS11240	*psaE*	photosystem I reaction center subunit IV
SGL_RS09810	*psaF*	photosystem I reaction center subunit III
SGL_RS17825	*psaI*	photosystem I reaction center subunit VIII
SGL_RS09805	*psaJ*	photosystem I reaction center subunit IX
SGL_RS02600	*psaK1*	photosystem I reaction center subunit PsaK 1
SGL_RS17240	*psaK2*	photosystem I reaction center subunit PsaK 2
SGL_RS17820	*psaL*	photosystem I reaction center subunit XI
SGL_RS04090	*psaM*	photosystem I reaction center subunit XII
		b6-F
SGL_RS07315	*petA*	apocytochrome f
SGL_RS13200	*petB*	cytochrome B6
SGL_RS07320	*petC1*	cytochrome b6-f complex iron-sulfur subunit 2
SGL_RS18155	*petC2*	cytochrome b6-f complex iron-sulfur subunit 1
SGL_RS10620	*petC3*	cytochrome B6
SGL_RS13205	*petD*	cytochrome b6-f complex subunit 4
SGL_RS10475	*petG*	cytochrome b6-f complex subunit 5
SGL_RS16365	*petM*	cytochrome b6-f complex subunit 7
		Photosynthetic electron transport
SGL_RS13610	*petE*	plastocyanin
SGL_RS13450	*petF*	Ferredoxin-1
SGL_RS06260	*petF, fdx*	ferredoxin

SGL_RS10530	*petF, fdx*	ferredoxin
SGL_RS12135	*petF, fdx*	ferredoxin
SGL_RS11630	*petH*	ferredoxin--NADP(+) reductase
SGL_RS05875	*petJ*	cytochrome c6
	PSII	
SGL_RS08090	*psb28-2*	photosystem II protein
SGL_RS18130	*psbA1*	photosystem II protein D1 1
SGL_RS01910	*psbA2*	photosystem II protein D1 2
SGL_RS10440	*psbA3*	photosystem II protein D1 2
SGL_RS14825	*psbB*	photosystem II CP47 reaction center protein
SGL_RS08200	*psbC*	photosystem II CP43 reaction center protein
SGL_RS08205	*psbD*	photosystem II D2 protein
SGL_RS16825	*psbD2*	photosystem II D2 protein
SGL_RS04530	*psbE*	cytochrome b559 subunit alpha
SGL_RS04535	*psbF*	cytochrome b559 subunit beta
SGL_RS07325	*psbH*	photosystem II reaction center protein H
SGL_RS12880	*psbI*	photosystem II reaction center protein I
SGL_RS04545	*psbJ*	photosystem II reaction center protein J
SGL_RS04440	*psbK*	photosystem II reaction center protein K
SGL_RS04540	*psbL*	photosystem II reaction center protein L
SGL_RS11735	*psbO*	photosystem II manganese-stabilizingpolypeptide
SGL_RS09330	*psbP2*	hypothetical protein
SGL_RS13125	*psbT*	photosystem II reaction center protein T
SGL_RS03265	*psbU*	photosystem II extrinsic protein
SGL_RS11935	*psbV*	cytochrome c-550
SGL_RS02070	*psbW*	photosystem II reaction center Psb28 protein
SGL_RS14050	*psbX*	photosystem II reaction center X protein
SGL_RS07800	*psbY*	photosystem II protein Y
SGL_RS11645	*psbZ*	photosystem II lipoprotein Psb27
	ATP synthase	
SGL_RS02675	*atpA*	ATP synthase subunit alpha
SGL_RS09705	*atpB*	ATP synthase subunit beta
SGL_RS02670	*atpC*	ATP synthase subunit gamma
SGL_RS02680	*atpD*	ATP synthase subunit delta
SGL_RS09710	*atpE*	ATP synthase epsilon chain
SGL_RS02685	*atpF*	ATP synthase subunit B
SGL_RS02690	*atpG*	ATP synthase subunit b'
SGL_RS02695	*atpH*	ATP synthase subunit C
SGL_RS02700	*atpI*	ATP synthase F0F1 subunit A
	Carbon fixation	
SGL_RS13425	*rbcS*	ribulose bisphosphate carboxylase small chain
SGL_RS09950	*rpe*	ribulose-phosphate 3-epimerase
SGL_RS07215	*gap1*	glyceraldehyde-3-phosphate dehydrogenase
SGL_RS18020	*xfp*	phosphoketolase

SGL_RS11420	*fbpII*	fructose 1,6-bisphosphatase
SGL_RS11625	*prk*	phosphoribulokinase
SGL_RS13415	*rbcL*	ribulose bisphosphate carboxylase large chain
SGL_RS13180	*tpi*	triose-phosphate isomerase
SGL_RS05660	*tktA*	transketolase
SGL_RS11290	*ppc*	phosphoenolpyruvate carboxylase
SGL_RS11980	*pgk*	phosphoglycerate kinase
SGL_RS09170	*fbpI*	fructose 1,6-bisphosphatase
SGL_RS14580	*rpiA*	ribose-5-phosphate isomerase
SGL_RS17045	*gap2*	glyceraldehyde-3-phosphate dehydrogenase
SGL_RS13385	*fbaA, fda*	fructose-bisphosphate aldolase
SGL_RS14885	*mdh*	malate dehydrogenase
SGL_RS11345	*fda*	class I fructose-bisphosphate aldolase
Phycocyanin biosynthesis		
SGL_RS07880	*hemA*	glutamyl-tRNA reductase
SGL_RS13390	*hemL*	glutamate-1-semialdehyde 2,1-aminomutase
SGL_RS09590	*hemB*	delta-aminolevulinic acid dehydratase
SGL_RS07710	*hemC*	hydroxymethylbilane synthase
SGL_RS12770	*hemD*	uroporphyrin-III synthase
SGL_RS16675	*hemE*	uroporphyrinogen decarboxylase
SGL_RS03345	*hemF*	coproporphyrinogen III oxidase
SGL_RS05455	*hemN*	coproporphyrinogen III oxidase
SGL_RS10380	*hemN*	coproporphyrinogen III oxidase
SGL_RS03055	*hemJ*	protoporphyrinogen IX oxidase
SGL_RS15155	*hemH*	ferrochelatase
SGL_RS03350	*ho1*	Heme oxygenase 1
SGL_RS10385	*ho2*	Heme oxygenase 2
SGL_RS15810	*pcyA*	phycocyanobilin:ferredoxin oxidoreductase
**Cyclic electron flow**		
SGL_RS12625	*ndhD1*	NAD(P)H-quinone oxidoreductase chain 4-1
SGL_RS03225	*ndhD2*	NAD(P)H-quinone oxidoreductase chain 4-2
SGL_RS06290	*ndhD3*	NAD(P)H-quinone oxidoreductase subunit D4
SGL_RS06295	*ndhF3*	oxidoreductase
SGL_RS06050	*pgr5*	hypothetical protein
**Nitrogen assimilation**		
SGL_RS03810	*amt1*	ammonium transporter
SGL_RS15695	*amt2*	ammonium transporter
SGL_RS16895	*amt3*	ammonium transporter
SGL_RS06595	*nrtA*	nitrate transporter NrtA
SGL_RS06590	*nrtB*	nitrate transport permease nrtB
SGL_RS06585	*nrtC*	nitrate transport ATP-binding protein NrtC

SGL_RS06580	*nrtD*	bacitracin ABC transporter ATP-binding protein
SGL_RS06575	*narB*	nitrate reductase catalytic subunit
SGL_RS14775	*nirA*	ferredoxin--nitrite reductase
SGL_RS11925	*glnN*	glutamine synthetase
SGL_RS04825	*gifA*	glutamine synthetase inactivating factor
SGL_RS04030	*gifB*	glutamine synthetase inactivating factor
SGL_RS13880	*trpG*	glutamine amidotransferase
