# Supplementary figures and images for: Different Regulatory Modes of Synechocystis sp. PCC 6803 in Response to Photosynthesis Inhibitory Conditions
Source: mSystems. 2021 Dec 7;6(6):e00943-21. doi: 10.1128/mSystems.00943-21 (PMC8651088; doi:10.1128/mSystems.00943-21)

**A**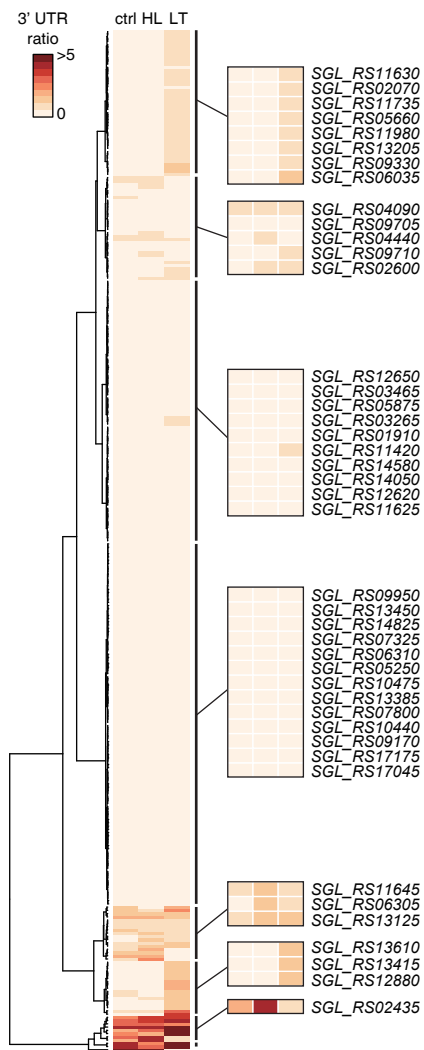**B**

■ RPF reads (log<sub>2</sub>)

■ RNA reads (log<sub>2</sub>)

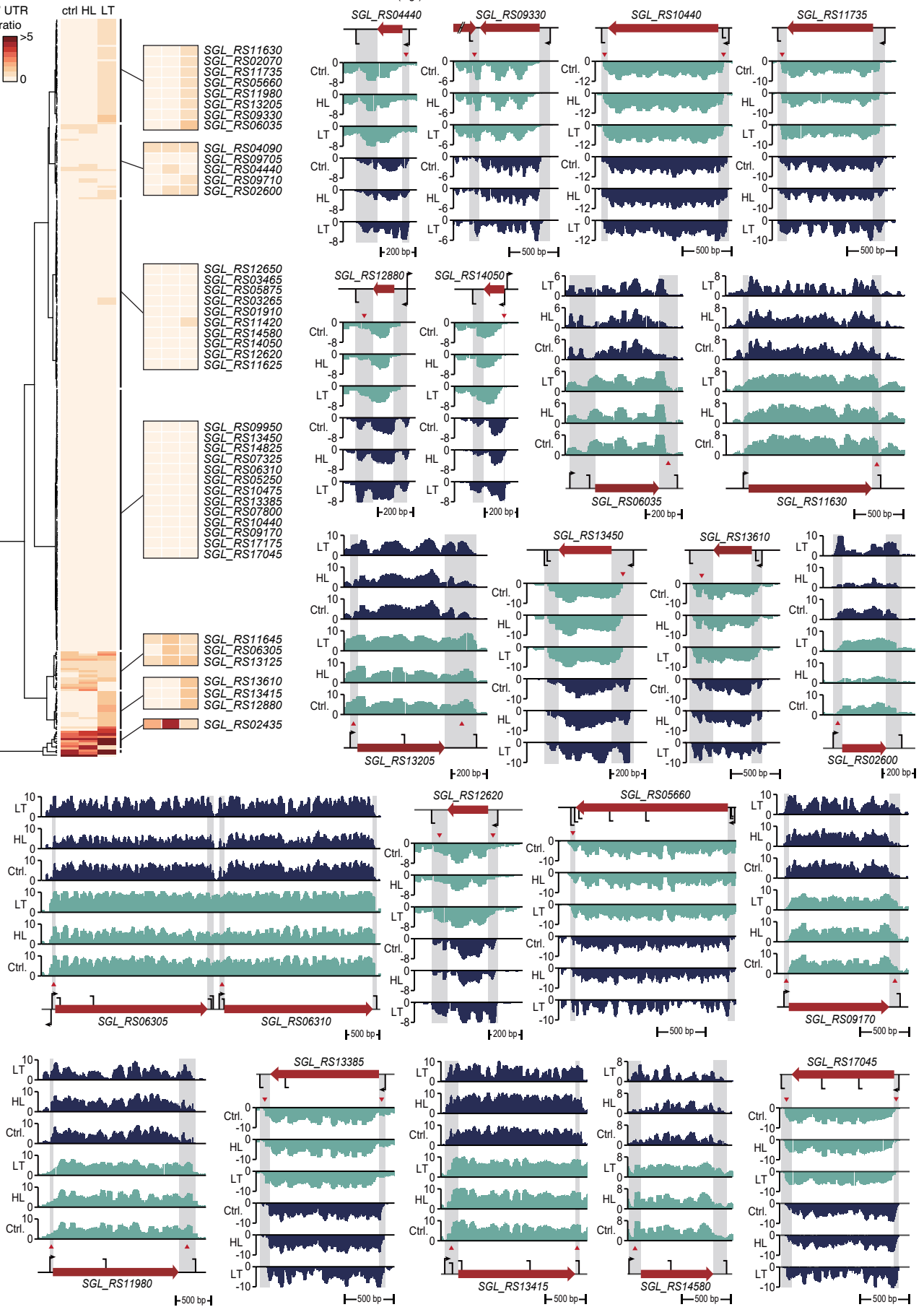

Supplement: FIG S5 [file msystems.00943-21-sf005.pdf]
