# Supplementary material for: Different Regulatory Modes of Synechocystis sp. PCC 6803 in Response to Photosynthesis Inhibitory Conditions
Source: mSystems. 2021 Dec 7;6(6):e00943-21. doi: 10.1128/mSystems.00943-21 (PMC8651088; doi:10.1128/mSystems.00943-21)
Supplement: TABLE S7 [file msystems.00943-21-st007.pdf]

Table S7. Primers used for quantitative real-time PCR.

Gene		Sequence (5'→3')	Product length (bp)
SGLS07010	Forward	TCACTTTAGTCCTGGGGACA	106
	Reverse	CTGAATGGGAATGTAGGCGG	106
SGLS17175	Forward	TATCTAGACCGGAACGCCAT	104
	Reverse	ACGATGGTGGCCGAATTG	104
SGLS01910	Forward	GGTGACCTCTACCAACAACC	103
	Reverse	GGCGATGAAGGCAATGATGA	103
SGLS06310	Forward	GGCGTATTTGGTACGGGATT	107
	Reverse	GATGTGTCCAAAGTGGGAGG	107
SGLS09805	Forward	TCCTTTTTGTCAACTGCTCCG	103
	Reverse	GGTGGAAAAGAAGATCGGGA	103
SGLS02600	Forward	AGTTGCTGGGGTGATGATTG	101
	Reverse	AGTTAGAGGAAGGCATGGGG	101
SGLS10230	Forward	ACGTTGTACCCGCTACCATC	105
	Reverse	CGATCGTAAGGGTTTTACCCA	105
SGLS06595	Forward	CGCTATTGGAGGAGTGGTTC	110
	Reverse	CCCCCTCGACTAAAGGACTA	110
